# ‘Does that mean you will be violent?’: A qualitative exploration into Autistic women’s experiences of relationships with lecturers at university

**DOI:** 10.1177/13623613241264887

**Published:** 2024-07-26

**Authors:** Sophie Phillips

**Affiliations:** The University of Sheffield, UK

**Keywords:** autism, relationships, stigma, women

## Abstract

The experiences of Autistic^1^ women students at university are seldom explored. In this paper, *women* refers to anyone who self-identifies as a woman. Eleven Autistic women students participated in artefact creation and semi-structured interviews exploring their experiences with university lecturers. All data centred on their opinions and lived experiences. Five participants were undergraduate students and six participants were postgraduate students. Data were analysed by thematic analysis and data was categorised into two themes. These themes were: ‘Lecturer Impact’ and ‘Autism Awareness and Acceptance’. ‘Lecturer Impact’ centred around both positive relationships participants had at university with their lecturers, and how they navigated ingrained stigmas about autism that some lecturers held. ‘Autism Awareness and Acceptance’ included the ways in which knowledge about autism in women and a reduction in stigma could be achieved at university, primarily by incorporating the voices of Autistic women students. The findings suggest that Autistic women students’ positive relationships with lecturers are key to success. This was supported by participants wanting their opinions and lived experience to be valued particularly to assist with raising a university culture of autism acceptance. An inclusive environment at university, including Autistic women students is needed to improve positive experiences at university.

**Lay abstract:**

It is documented that more Autistic woman are becoming university students, but their experiences are not often explored. Eleven Autistic women students made artefacts (such as writing, paintings or poems) and took part in interviews to share their experiences at university. Five participants were undergraduate students and six participants were postgraduate students. The artefacts and words participants shared were looked at closely and some common themes were decided. How lecturers talk to Autistic women students was looked at. Participants said they were lucky if they met helpful lecturers. Also, autism acceptance was explored. Overall, how lecturers treat Autistic women at university is very important. Also, Autistic women students’ opinions about making university a more welcoming place need to be listened to.

## Introduction

Before turning to my study, I explain my positionality in relation to the research. Cage and Howes (2020) highlighted the importance of acknowledging and discussing the author’s positionality in qualitative research in order to ensure the reader has a sense of the potential reasoning behind methodical and analytic decisions. An author’s positionality may also indicate their motivations behind a study. Oliveira (2019) emphasised that feminist researchers bring their own culture, understandings and identity to research, and need to consider how these impact the meanings being placed on data. My interest in researching Autistic experiences within the Higher Education sector was initially influenced from my own predominantly negative experiences at university, prior to receiving an autism diagnosis in the late stages of my undergraduate study. Undertaking postgraduate study with the ‘new’ identity as an Autistic woman highlighted to me firsthand both challenges and positives of being Autistic at university. I undertook this research to add to the growing body of literature exploring the Autistic experience at university, that ultimately aims to create change around the inclusivity of Autistic people in Higher Education.

I support the principles of the neurodiversity movement (den Houting, 2019), both for myself and my participants. I primarily base my research on disability feminist principles (e.g. Garland-Thomson, 2002) from my positionality as an Autistic woman, . I bring my own lived and subjective experiences, both positive and negative, of being an Autistic woman in a society that has historically marginalised women and in particular Autistic women.

In the academic year 2021–2022, 6260 women university students in the United Kingdom declared they had a ‘social communication disability/autism spectrum disorder’ (Higher Education Statistics Agency [HESA], 2023). Every academic year since 2014/2015 HESA suggest this figure has increased by approximately 1000 students (HESA, 2023). These figures do not include women who declare more than one disability label (e.g. a woman who is autistic and ADHD). Despite increasing numbers of women studying at university declaring they are Autistic, little is known about their experiences. This is not to say that Autistic women (or women who had unlabelled Autistic traits) did not attend university when statistics were reportedly lower, but considering these stark statistics are now printed and accessible, action needs to be taken to ensure this demographic are not ignored in research. It is also vital to focus research on Autistic adults, as they have been regularly documented in research to have poor outcomes compared to neurotypical adults, such as less independence, higher rates of mental health difficulties and poor job prospects (e.g. Henninger & Taylor, 2013; Howlin & Magiati, 2017; Lee et al., 2023). These poorer outcomes may be due to society’s lack of adherence to the social model of disability and society’s adherence to neurotypicality. In relation to Autistic people, Dwyer (2022) advocated that embracing the social model (that disability consists of both impairment and environment) may reduce the dominant societal perception of autism, that all Autistic traits that deviate from the norm should be reduced in an individual. Considering these poor outcomes, and the effect others can have on an Autistic person, more aspects of the adult Autistic experience need exploration.

## Autism in women

The Autistic experience can be gendered. The gender diagnosis ratio has been suggested to be as low as 1.8:1 (Mattila et al., 2011). Autistic women may not display the same characteristics as Autistic boys and cis-gendered men, or mask their Autistic traits and therefore are less frequently identified and diagnosed (Pearson & Rose, 2021). This ratio suggests that autism occurs frequently in girls and women and therefore the impact of autism on girls and women needs to have the same level of research as that which has traditionally focused on men. Hoyt and Falconi (2015) argue that there needs to be an increase in research into the Autistic experiences of women, because being biologically female attracts particular health and mortality risks. However, I believe this may be difficult considering that Autistic women are viewed negatively, or routinely ignored, in society. This is because the experiences of Autistic women are repeatedly under-represented within research (for example, Hoyt & Falconi, 2015; Milner et al., 2019; Seers & Hogg, 2021). Therefore, contributing to the research field by explicitly documenting the experiences of Autistic women at university may help to reduce common societal gender stereotypes surrounding autism (Milner et al., 2019).

## Autism at university

Much research focuses on the impact of neurotypical students’ relationships with lecturers in helping or hindering their enjoyment and achievement in academia (Guzzardo et al., 2021; Hagenauer & Volet, 2014; Yale, 2019). However, research seldom focuses on the interactions of lecturers and Autistic students. There are some notable exceptions. Most recently, Johnson et al. (2023) interviewed 12 Autistic university students’ about how they viewed themselves academically. They found that most students had a positive academic self-concept, but that it was affected by the way reasonable adjustments were implemented by staff. They concluded that universities need to further explore training of academic faculty in order to improve how Autistic students are accommodated and that later diagnosed Autistic women’s experiences needed to be focused on more frequently in research. Bailey et al. (2020) surveyed 42 Autistic university students and interviewed 23 of them regarding their social experiences at university. The majority of their participants identified as male. They concluded that being more socially engaged with peers at university was associated with more positive experiences, such as better well-being. However, contrary to what might be expected they also highlight that Autistic students who are having more positive experiences, such as being socially engaged with peers or being part of university societies are more likely to have negative interactions with professors, particularly in relation to accessing accommodations. Bailey et al. (2020) suggested that this might be due to lecturers perceiving they do not need as much support if they appear to be enjoying university, or students feeling empowered and aware of their rights. Therefore, although an Autistic student may be socially connected with peers and thus more involved in university, it does not negate that they may have negative experiences non-Autistic peers are unlikely to be subjected to. It is interesting to consider these findings in light of the majority of their participants identifying as men, particularly as societal stereotypes of autism tend to focus on how autism is portrayed in boys and men such as being very good at mathematics and not enjoying any social interaction.

Scott and Sedgewick (2021) highlighted the importance of good relationships at university with both peers and staff in order to aid the positive mental health of Autistic students. Scott and Sedgewick (2021) highlighted that ‘Autistic voices regarding experiences of university support remain strikingly absent’ (p. 3). They interviewed 12 Autistic students about factors surrounding mental health and support at university. Scott and Sedgewick (2021) documented that although results showed three themes (relationships, independence and support) they were overarchingly linked by relationships. They explained this link as fundamental, as when relationships were supportive university went well, but when they involved stigmatising attitudes university presented more difficulties. Scott and Sedgewick (2021) presented mixed responses from participants regarding relationships. Some participants noted that some academic staff were knowledgeable and accepting about autism, enabling them to feel confident in asking for adjustments and accommodations. However, other participants felt staff they had interacted with had a lack of autism awareness and were less accommodating to their needs. These students thus felt stigmatised and unwilling to seek support for any difficulties they had at university. Other research has highlighted the stigma Autistic students face at university (Gillespie-Lynch et al., 2015; Perry et al., 2022). Stigma refers to a combination of prejudice and discrimination, which may cause the oppressed group to be restricted in what they can do (Link & Phelan, 2001). Others’ perceptions of a person can either help or hinder a person to succeed (Richard & Hennekam, 2021), and therefore it is important to investigate how Autistic students are considered and whether perceptions need to be addressed. These findings including from Bailey et al. (2020), Johnson et al. (2023) and Scott and Sedgewick (2021) highlighted that stigma towards Autistic students at university from academic staff still persists, whether it stems from a lack of understanding about autism and its heterogeneity or an unwillingness to provide support. Although there is evidence to suggest that some Autistic students are well received and supported by academic staff and peers this seems to be the exception rather than the norm (Cage & Howes, 2020). Therefore, Autistic students are possibly having to educate other students and lecturers through self-advocacy while trying to navigate university themselves, which can be exhausting and be overwhelming (Lei & Russell, 2021). Thus, creating extra labour for themselves in situations where how people react may be not what was hoped for.

Research specifically exploring the attitudes of non-Autistic university students towards Autistic students also tends to be negative. White et al. (2017) explored university students’ knowledge of autism and attitudes towards Autistic people and compared their results to a similar study they conducted five years earlier, where they highlighted that participants held negative attitudes towards Autistic people and had poor knowledge about autism. Although these participants demonstrated greater knowledge than the non-Autistic students from the previous study, many continued to hold similar negative attitudes towards Autistic peers. However, knowing an Autistic person appeared to be associated with less negative attitudes held by the non-Autistic participants. Wang et al. (2023) investigated the opinions of 712 non-Autistic university students about their attitudes towards Autistic students, using non-gendered vignettes. They documented mixed findings. Participants who were told the character in the vignette was Autistic tended to show more favourable attitudes towards Autistic people. Most notably they suggest that in order for an Autistic person to be accepted at university, it is their responsibility to disclose their diagnosis rather than the onus being on non-Autistic students to simply be inclusive to everybody. Although both of these studies are primarily looking at how non-Autistic students perceive Autistic students, they both describe the different aspects of the participants’ identities but consider Autistic people as a homogeneous group. Therefore, the researchers recognise the diversity and unique characteristics of the non-Autistic participants but neglect doing this for the Autistic people. Autistic people (like non-Autistic people) have diverse life experiences and perspectives as well as intersectional identities (e.g. race and sexuality) that makes it inaccurate to present them as being homogeneous (Crenshaw, 1989). Generally negative attitudes towards any Autistic person needs to be changed; however, more nuanced understandings of stigma in relation to other intersectional identities may be beneficial in developing stigma tackling initiatives.

### Current study

The findings from these studies provide essential knowledge about the importance of relationships for Autistic people at university and the stigma that they may receive. Considering the growing numbers of Autistic women attending university and the gendered stereotypes of autism that society holds, it is important to explore the experiences of their relationships and stigma Autistic women students may be subjected to. Experiences of Autistic women are routinely neglected from research (Taylor & DaWalt, 2020). This research can further knowledge about the gendered experience of autism and explore ways to reduce stigma towards Autistic women and improve relationships. This current study highlights a particular need for increased acceptance towards Autistic women in Higher Education, as they face higher rates of marginalisation than their male counterparts (North, 2023). The aim of this study was to build on current research about the Autistic experience at university, with specific focus on understanding interactions with lecturers and Autistic women.

## Methods

### Research focus

This study draws on empirical findings from an exploratory qualitative research study investigating Autistic women’s experiences of university. I focus this paper on presenting participant experiences about the impact of lecturers’ opinions and behaviour.

### Participants

Eleven Autistic women participated in creating artefacts (examples that were created included paintings, poems, photographs and descriptive writing) and semi-structured online interviews in early 2020. Inclusion criteria were (1) participants had to identify as a woman, (2) participants self-identified as Autistic and (3) participants were at the time of being recruited studying at university in the United Kingdom. Autism diagnosis can be viewed as a privilege and inaccessible to many, therefore self-identification of autism was viewed as acceptable (Giwa Onaiwu, 2020; Leedham et al., 2020). The research was restricted to participants at UK universities so that it could be streamlined more easily as disability support, on paper, is broadly similar across the United Kingdom. Given the open-ended nature of the methods, specific demographic information such as ethnicity and disability were only partially revealed during data collection at the preference of the participant. Table 1 shows the demographic information that was collected. Ethical approval was granted from The University of Sheffield.

**Table 1.** Demographical data.

Participant name (* denotes a pseudonym)	Level of Study	Country of Study	Ethnicity	Other Disability Labels	Number of Artefacts	Type of Artefacts	Type of Skype Interview
Billy*	Postgraduate	Wales	White British	Mental Health Difficulties	3	Photo and Written Description	Text
Cassy*	Postgraduate	Wales	White British	Dyslexia	3	Sketches	Voice
Katie*	Postgraduate	England	Not Declared	ADHD	3	2x Artwork 1x Description	Voice
Kim*	Postgraduate	England	White British	None	3	1x Artwork 1x Photo	Video
Jess*	Undergraduate	England	Not Declared	None	2	Poem	Text
Lilly*	Undergraduate	Northern Ireland	Not Declared	None	3	2 x Paintings 1 x Poem	Text
Megan	Undergraduate	England	Not Declared	Sensory Processing Disorder and Auditory Processing Disorder	2	Written Description	Text
Poppy	Postgraduate	England	Not Declared	ADHD	1	1x Painting	Voice
Sarah*	Postgraduate	England	White British	Prefer not to say	2	2x Artwork	Video
Sophia*	Undergraduate	Scotland	White British	None declared	3	2x Artwork and Description 1x Description	Video
Sophie	Undergraduate	England	White British	None declared	3	2x Sketch and Description 1x Description	Video

### Methodical approach

This study was situated within a Feminist Disability Studies paradigm, to amplify the voices and opinions of the participants (Garland-Thomson, 2002). First the artefacts were analysed based on Culshaw’s (2019) analysis of collages, by considering the descriptive elements (how it was produced) of the artefact, the analytical features (e.g., compositional context and significance of colour) and then my interpretation (generating obvious and then alternative readings of the image). My analysis of the artefact (as an autistic women researcher) was then added to the participant’s own description of their artefact. Then, Inductive thematic analysis was used to analyse the interview data. Braun et al. (2022, p. 431) highlight that their explanation of thematic analysis is a ‘springboard’ and ‘invitation’ rather than a dictatorial set of instructions to follow. I carried out Braun and Clarke’s (2006) six steps of analysis. The artefacts and their descriptions were also thematically analysed. No other researchers were involved in the coding process. Participants were asked for their comments about how I had coded their transcripts to ensure that I had represented their views as they wished.

### Procedure

The study was advertised on X (formerly Twitter), where interested individuals were directed to email from a university email address for further information. This was to attempt to reduce the likelihood of fake participants. Participants’ were asked to self-identify as a woman and as Autistic. Pellicano et al. (2024) highlighted that the likelihood of participants pretending to be Autistic to participate in studies for financial gain is higher in qualitative online research. I did not inform participants of how much they would be financially compensated until they had completed their first artefact, in order to deter any fraudulent participant. In the study advert I was open about my identity as an Autistic woman. Some participants said that they would not have taken part in the study if I had not disclosed that I was Autistic. X was used to reduce the need to engage with gate-keepers and for convenience to reach potential participants quickly. Participants were provided an information sheet about the study documenting the outline of the study, that the study may potentially provoke negative emotions, who to contact for support and how to withdraw from the research. They were also asked if they wanted their artefacts and words attributed to a pseudonym or their real name. Participants were then asked to sign a consent form to demonstrate agreement.

Participants were asked to create up to three artefacts documenting experiences at university that they felt were impacted by being Autistic (either positively or negatively) in any medium they had access to at home that could be emailed (such as a photographed painting). Participants were deliberately not given any further instructions in order to promote creativity. Each participant was given up to 8 weeks to create their artefacts and they were emailed every 2 weeks within this time period to ensure they still wanted to participate in the project and ask if they had any difficulties. Each participant then took part in an online semi-structured interview. Interviews were conducted on Skype (a software that enables online video calls) and participants had the choice of whether to participate via video, voice or text (see Table 1: Demographical Data). The interviews were recorded, in order to aid transcription by hand, after which the video recordings were deleted. The questions focused on: (a) the creation of their artefacts, (b) the meanings behind them, (c) the barriers and enablers of being an Autistic woman student and (d) how the university experience could be changed to enhance their experience. Questions and potential follow-up topics were emailed to participants a week before the interview. Participants were then thanked for their time and given a £20 voucher.

### Community impact statement

The project was designed by myself, an Autistic adult, as part of my PhD research. No additional further community involvement occurred in the design of the study.

## Findings

The data presented forms part of a larger study. Within the larger study, data were grouped into three themes. In this paper I specifically focus on one theme, in which I explored reflections on the university environment. Data discussed here were grouped into two topics: ‘Lecturer Impact’ and ‘Autism Awareness and Acceptance’. I discuss each of these topics in turn, with quotes from participants. Excerpts and descriptions of participants’ artefacts are also included. These results formed part of a larger research project. The other two themes of the larger project were ‘Perceptions’ and ‘Exposing the Postgraduate Autistic Student’.

### Lecturer impact

#### Lecturers’ impact

Several participants felt privileged to have encountered lecturers that had been supportive and helpful towards them. They attributed these good experiences with lecturers to luck and privilege, but not accessibility or right. In order to be lucky however, an Autistic person may need to feel safe enough to disclose that part of their identity. Some participants highlighted positive experiences they had at university, due to attitudes and extra effort of lecturers to be accommodating, for example Kim said

My two supervisors have bent over backwards for me . . . nothing was too big to ask of them and whenever autism has come into it, the same response has been given and they are very very understanding . . . So, I do feel very very privileged to be in a university that is so supportive.

Sophie and Cassy described being lucky, ‘I am lucky because university is kind to me but I can imagine if it was not that it would be really difficult’. (Sophie) and ‘I am very lucky that our head of admin and our learning and teaching coordinator is very aware of my sort of shortcomings, as it were, and she’s very good at helping me manage that’. (Cassy).

How participants described being positively supported suggests they were aware that other Autistic people may not have similar experiences. In contrast, some students implied that because they did not have a formal diagnosis of autism, they were not able to be supported in the way they felt they needed to be. Thus, they had no chance of being able to be lucky and receive good support. Poppy said, ‘I feel like, in a way, there is a lot more support there for people who are like openly Autistic. The thing is you probably still have to fight but at least you have got a starting point’. Katie highlighted, ‘Something that kind of triggered my supervisor to tell me that I should probably go for a diagnosis because she was going to have to change the way she gives me feedback’. This suggests that luck may be based not only on university structures and the lecturers within, but also on an Autistic’s person’s willingness to disclose their diagnosis and be able to advocate for themselves to get support. In addition, luck may be based on understanding what is needed to succeed academically and if some support is provided, taking it, and continuously advocating for it to continue which in itself is privilege.

Some participants encountered lecturers basing interactions on ingrained stereotypes when they disclosed a diagnosis. Sophia encountered negative stereotypical attitudes during an interview for a university course, which she documented in a piece of descriptive writing she created for the project,

Towards the end of the interview, I spoke up and explained that I had just been diagnosed [with autism]. I expressed that I was unsure what this meant in terms of education, but I was interested in returning to study and to try my best. ‘Does that mean you will be violent?’ That’s all he said. And that’s all it took. A Professor, an arguable intellectual adult man asking me if I was going to attack others. (Sophia, excerpt from her artefact, ‘The Interview (Returning to Education)’).

Having a formal diagnosis of autism may therefore allow a student to more easily navigate bureaucracy associated with gaining support, but not the opinions and stereotypes of others. Pearson and Rose (2021) argue that Autistic people are regularly shown or told that they are abnormal or impaired. This can be internalised and damaging to an individual’s self-esteem and confidence in their abilities. Overall, how lecturers engaged with Autistic students had a big impact on how they felt about university. Some participants attributed positive relationships with lecturers to luck, whereas others felt they were unable to access such support due to university policies. It is important to note however that what luck looks like, and the privilege that may proceed this, might be different for every autistic woman. This suggests that lecturers need to promote inclusive atmospheres but also be supported by the policies and expectations of universities.

#### Autism awareness and acceptance

Participants were keen that awareness of autism needs to improve, but lecturers and students need to not only be aware of autism, but also accept Autistic people. However, participants did not think their voices were being listened to or taken seriously and voiced a strong desire to be listened to, both in society and within the university setting, especially when they wanted to advocate for change. In addition, practical changes may help to break harmful stereotypes that people may hold about autism and challenge the usually negative thinking around it.

Two participants how they felt lecturers and other students at university simply did not understand what autism was. This made it difficult for them to have positive experiences at university as they did not feel understood by the people around them, Kim said, ‘I think a lot of it is about education. The issues I have had with lecturers etc have just been about ignorance’. and Lilly voiced, ‘Some lecturers and many of my fellow students are not very autism-aware’. With regard to raising awareness and acceptance, that is evidently needed from participants’ experiences, opinions about what may make effective training on autism at university were mooted: ‘I think more awareness of how autism looks genuinely would make a difference’. (Cassy) and ‘I think it is just understanding different needs because there is so much stuff that is not talked about with autism’. (Sophie).

It was highlighted that changing ingrained stereotypes and perceptions may be difficult as society appears to already know what autism is, has contact with Autistic people, and thus has fixed opinions of it which may be difficult to change. Kim suggested,

I am very lucky that autism is something that you know, everyone’s got an Autistic cousin or godparent or something these days . . . people do not look at me as if I have got three heads, but they do not necessarily fully understand what it is and again, they have those stereotypes. So breaking stereotypes via a campaign, I think would be something that I would really like to see universities do.

Whereas Jess said, ‘I think speaking more openly about Autistic women. I think because there is this false and harmful notion that only men are Autistic. We need to highlight Autistic women’.

However, for Autistic people to be at the forefront of breaking stereotypes at university and helping to create change, they need to be heard by others and their voices accepted. Billy and Kim both highlight that Autistic people want to be listened to but are not always. They expressed, ‘Listen to students with firsthand knowledge. The experts [Autistic people] are all out there, all they need to do is ask and a willingness to listen goes a long way’. (Billy) and ‘[Autistic] people just want to be listened to and not put in a box’. (Kim). Overall, participants were hopeful that there is capacity for improvement in inclusivity and believe that this could be done through raising awareness. A combination of considering greater awareness and training, listening to and acting on what Autistic students have to say and striving to provide inclusive events that do not rely on Autistic stereotypes from non-Autistic people maybe key to ensuring Autistic students are not disadvantaged at university.

## Discussion

The aim of this study was to explore the experiences of Autistic women at university, with relation to their experiences with lecturers. In this paper, I have attended to the themes of ‘Lecturers Impact’ and ‘Autism Awareness and Acceptance’ that were prominent in the data. This study contributes to understanding the experiences of Autistic women at university.

First, data revealed the impact of relationships that participants had with lecturers. Several participants felt privileged to have encountered lecturers that had been supportive and helpful towards them. Some Autistic students attributed good experiences with lecturers to luck and privilege. Second, data illuminated participants’ experiences of advocating for change and educating others about autism. Participants highlighted the need to be listened to and included in initiatives to improve autism acceptance. It was also evident that harmful stereotypes towards Autistic women still exist and that negative thinking about autism needs to change.

Reporting about lecturer relationships was mixed, as some participants reported feeling supported by lecturers, whereas others had had poor experiences. For example, Kim’s experienced being well-supported by her supervisors, but Sophia received stigmatising comments. Kim and Sophia’s differing experiences may have been down to a variety of factors, for example, Sophia’s professor suggesting that she might be ‘violent’ demonstrates an ingrained societal stereotype associated with autism. Although, it may not be a stereotype typically associated specifically with Autistic women, non-gendered negative stereotypes about autism can still be damaging. This is compared to Kim’s supervisors treating her as an individual rather than solely by stereotypes.

Some recent qualitative research into Autistic people’s experiences at university has included the concept of luck (for example, Lei & Russell, 2021; Scott & Sedgewick, 2021). These studies do not explore why participants state they are lucky and the impact of whether good support is effectively a lottery. In the current study some participants described themselves as ‘lucky’ or ‘privileged’ to be so well supported, which perhaps suggests that they are aware or believe that not all Autistic students can access such good support. Participants were not specifically asked about luck in the interviews, but rather asked about the supports they received at university. This begs the question of why participants should feel lucky to be supported, rather than it being assumed it will happen. In addition, it is questionable whether an Autistic person’s success at university is in part determined by luck, rather than by a right to access and accommodations. This indicates maybe there is some internalised stigma surrounding how participants perceive the university to view them.

In contrast, other participants implied that because they did not have a formal diagnosis of autism, such as Katie’s experiences with feedback, they no chance of being able to be lucky and receive good support. They reported they could not access formal support and lecturers felt unable to support them without a diagnosis. This supports the work of Hens and Langenberg (2018) who suggested that a formal diagnosis of autism can lead to access to support. A formal diagnosis of autism can be seen as a privilege particularly for women due to gender difference in autism (Lai & Baron-Cohen, 2015) or being able to present as non-Autistic (Hull et al., 2017). This is particularly important to consider, as women tend to be diagnosed with autism at a much older age than men, which may mean they are unable to access formal support at university (Leedham et al., 2020).

Some participants expanded further on the privilege of diagnosis or willingness of lecturers to provide support. For example, Poppy highlighted that the onus is on the Autistic person to first get diagnosed and second to disclose this and advocate for their individual needs. This is similar to the argument of Bailey et al. (2020) who argued that Autistic students need to be taught self-advocacy and assertive communication skills to be able to self-advocate and challenge discriminatory behaviour, to assist with any inclusion training given to lecturers. This suggests that luck is not only based on university structures and the lecturers within, but also on an Autistic’s person’s willingness to disclose their diagnosis and be able to advocate for themselves to get support. Luck may also be based on other intersections of a person’s identity, which in turn act as a privilege. This may be particularly difficult for Autistic women who are already oppressed by patriarchal structures potentially through testimonial injustice, alongside autism stereotypes and stigma.

In considering how vital the attitudes of lecturers at university can be in acceptance, it is important to consider how this can be improved. In relation to improving the research landscape for Autistic people, Poulsen et al. (2022) argued ‘the heart of the message is, in part, about acceptance: embracing and valuing autism as part of the human spectrum’ (p. 3). However, data from this study suggest universities do not wholly follow this thinking, as luck and privilege may contribute, and thus autism awareness and acceptance are clearly still needed to ensure equity. Participants shared experiences emphasising how lecturers and other students are either ‘ignorant’ or ‘not very autism-aware’. These opinions complement research by Moriña Díez et al. (2015), who reported that disabled students believe lecturers create ‘more barriers than bridges’ (p. 155) with regard to inclusion, which may be due to a lack of awareness and acceptance of Autistic people.

Lack of awareness may be due to ignorance and may not be due to people actively wanting to perpetuate myths and stereotypes of Autistic people. However, the lack of education and awareness about autism like that is reported in this study that lecturers are particularly important. Waisman et al. (2023) argued that lecturers teaching university courses can influence students’ success at university, but that if lecturers have stigmatising attitudes towards Autistic students this can be hindered. Educating lecturers about autism might reduce this ignorance which could in turn stop Autistic people being subjected to misinformation and stereotypes. This is particularly important in relation to Autistic women, who already do not fit a normative stereotype of autism (Milner et al., 2019). The relationship between education quality and the level of ignorance about autism is debatable (Chown et al., 2023). They cautioned training is unlikely to change mindsets, however, awareness does need to increase to ensure Autistic women in particular are included and myths are not perpetuated.

Scott and Sedgewick (2021) and Johnson et al. (2023) concluded that staff should be trained to be confident in supporting Autistic students’ needs. Theoretically wanting people to be trained and what it could look like in practice are two different things. Just increasing a person’s knowledge about autism may not enable them to treat Autistic people better. Jones et al. (2021) concluded that training about autism may help to change some beliefs about autism but that ingrained stereotypes and prejudices may be harder to change. In addition, the link between increased knowledge about autism and reducing stigma towards Autistic people is contested (Kitchin & Karlin, 2022). Stronach et al. (2019) previously proposed that although knowing an Autistic person increased knowledge about autism, it had no effect on stereotypes and attitudes towards Autistic people.

In addition, many researchers (Botha & Gillespie-Lynch, 2022; Gernsbacher et al., 2018; Sarrett, 2018; Waisman et al., 2023) suggested that how knowledge about autism is produced and shared is important as they argue that Autistic people can be situated as not meeting an ideal of normalcy. Thus, if Autistic people are at the forefront of breaking stereotypes rather than solely being portrayed stereotypically by others, attitudes and ingrained stereotypes may change. For Autistic people to be at the forefront of breaking stereotypes at university and helping to create change, they need to be heard by others and their voices accepted. However, some participants in this study highlight the need to be ‘heard’ and listened to. This implies that they do not feel that their experiences and opinions are valued. This mirrors the thoughts of Gillespie-Lynch et al. (2022) who noted that Autistic people’s knowledge and expertise of autism are frequently overlooked, and that they are seldom involved in developing materials to increase autism awareness or educate others about it. Frost et al. (2019) argued that more autism awareness is not enough, but that society should strive for acceptance, inclusion and empowerment of Autistic people. Therefore, greater awareness and training, that does not rely on Autistic stereotypes is needed. Future trainings could be led by Autistic women students sharing their experiences including the ways in which they have been stigmatised, with the aim of highlighting how stigma can be reduced. In addition, it is also important to listen to and act on what Autistic students have to say so that they are not disadvantaged at university. Greater awareness and training may mean that all lecturers understand autism better and are able to provide good support to Autistic women at university, which is not based on an Autistic woman being lucky.

### Future directions

Future research could seek experiences of lecturers with regards to inclusion (or exclusion) of Autistic students, particularly in a ‘post’ Covid-19 world where neoliberalism at university is prominent. This could provide a more holistic view to ensure Autistic women students have supportive and positive university experiences. This is important to consider that the identity of lecturers, their own intersections and job roles are likely to inform how they respond to and support students. Lindsay and Fuentes (2022) said that academic workplaces are regularly described as challenging and toxic work environments. Thus, supporting students on top of a challenging workload and toxic environment may be difficult.

Future research could consider the importance of the attitudes of the whole university community towards Autistic women students and how lecturers can facilitate better knowledge and destigmatisation in non-Autistic students. Participants in this study also raised the issue that other students can hold stigmatised attitudes towards them. Many researchers (Anderson & Butt, 2017; Gardiner & Iarocci, 2014; Gelbar et al., 2015; Gillespie-Lynch et al., 2015; Gobbo & Shmulsky, 2012; Matthews et al., 2015; Obeid et al., 2015) have explored a variety of difficulties Autistic students can face at university. These studies consistently conclude that social stigma, combined with having to navigate social expectations of university present barriers for Autistic students. Therefore, student ignorance and stigma towards Autistic women students is likely to be as damaging as that from lecturers. If stigma from other students is reduced it may make navigating social expectations easier due to an acceptance from others. Lecturers may be able to influence and be part of this change.

## Limitations

The diversity of participants is a potential limitation in this research. This study used convenience sampling to attract participants. Potential participants were not asked to disclose other intersectionalities such as race or other disability labels. Malone et al. (2022) term this ‘scholarly neglect’. The impact of gender with respect to Autism may be different across the lines of additional intersectional difference. This is particularly important considering the concept of luck that some participants discussed. As mentioned in the Discussion Section luck is likely based on previous knowledge and privilege. A person’s intersectionalities, such as race may impact on prior access to knowledge. The aim of this study was exploratory, but further research should seek to look into how other intersections such as race impact the experience of Autistic women.

In the study, participants had to identify as a woman (thus including trans-women), which therefore excluded all men, but also people who identify as non-binary. The study could have appealed to a wider population by advertising for anybody who did not identify as a cis-gendered male. Further research could explore the impact of gender and autism with a wider population range.

Practically, using X (formerly Twitter) as a recruitment tool can be a limitation. Recently, X in particular has been subject to ongoing public ethical criticisms, potentially influencing whether people want to use the platform. It should be noted that I recruited participants via X in early 2020, prior to any concerns about the platform being publicly advertised. I used X to target a wide audience quickly and cheaply, however my recruitment poster will only have reached the population who use the platform. Herbell and Zauszniewski (2018) highlighted that different social media platforms tend to attract different age ranges of users and therefore suggest that in research not looking for a particular age demographic a range of social media platforms should be used. The aim of this research was on gathering experiences as an exploratory study and therefore sought to include any eligible person who saw my recruitment poster who was happy to share their experiences.

## Conclusion

This study aimed to explore Autistic women’s experiences of the university environment and the ways in which the environment could be improved for Autistic students. The findings brought both to the forefront how important relationships with lecturers and students’ feeling they have a voice that contributes to influencing change were. Participants cited a variety of reasons for this such as luck, privilege and lecturers’ knowledge of autism. Overall, ensuring a culture of acceptance and inclusion in university settings may reduce exclusion of not only Autistic women but also anybody else who feels they do not fit into an expected norm. This research adds to our understanding of how Autistic women experience the university environment and the factors that both help and hinder these experiences.

## Notes

1. Throughout this paper, I use identity-first language (using Autistic instead of ‘person with autism’) as that was the preference of all the participants in my study and is my personal preference as an Autistic researcher. Identity first language tends to be preferred by Autistic people; however, there are variations of preference within the wider autism community (Bottema-Beutel et al., 2021; Kenny et al., 2016).
